# Radiofrequency Induction Heating for Green Chemicals Manufacture: A Systematic Model of Energy Losses and a Scale-Up Case-Study

**DOI:** 10.1021/acsengineeringau.4c00009

**Published:** 2024-07-25

**Authors:** Jonathan P. P. Noble, Simon J. Bending, Alfred K. Hill

**Affiliations:** ^†^Centre for Sustainable and Circular Technologies, ^‡^Department of Chemical Engineering, ^§^Department of Physics, University of Bath, The Avenue, Claverton Down, Bath BA2 7AY, United Kingdom

**Keywords:** Radiofrequency heating, induction heating, energy efficiency, energy loss model, induction reactor

## Abstract

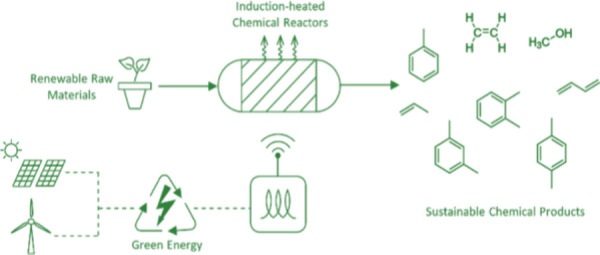

Radiofrequency (RF) induction heating has generated much interest for the abatement of carbon emissions from the chemicals sector as a direct electrification technology. Three challenges have held back its deployment at scale: reactors must be built from nonconductive materials which eliminates steel as a design choice; the viability of scale-up is uncertain; and to date the reported energy efficiency has been too low. This paper presents a model that for the first time makes a comprehensive analysis of energy losses that arise from RF induction heating. The maximum energy efficiency for radio frequency induction heating was previously reported to be 23% with a typical frequency range of 200–400 kHz. The results from the model show that an energy efficiency of 65–82% is achieved at a much lower frequency of 10 kHz and a reactor diameter of 0.2 m. Energy efficiency above 90% with reactor diameters above 1 m in diameter are predicted if higher voltage radio frequency sources can be developed. A new location of the work coil inside of the reactor wall is shown to be highly effective. Losses arising from heating a steel reactor wall in this configuration are shown to be insignificant, even when the wall is immediately adjacent to the work coil. This analysis demonstrates that RF induction heating can be a highly efficient and effective industrial technology for coupling high energy demand chemicals manufacture electricity from zero carbon renewables.

## 1 Introduction

Carbon emissions from energetically intensive industries such as steel, cement and chemicals are hard to abate (HTA) and account for around 23% of the global total.^1^ In the chemicals sector, green hydrogen and green electrification are often proposed as solutions. Hydrogen can substitute as both fuel and feedstock, but there are important challenges to its deployment. Electrolyser manufacturing capacity must increase by a factor of 6,000–8,000 to deploy on a global scale^2^ and green hydrogen production incurs significant energy loss in its generation and compression. Conversion efficiency for electrical power to delivered compressed hydrogen is around 60%, much lower than 75–90% which is typical for battery-electric based systems.^3^ Electrification has a significant energy efficiency advantage over hydrogen for energy intensive applications in a fixed location (where hydrogen is not needed as a reagent).

Energy efficiency is a critically important parameter for any energy intensive application and it would be a key part of a techno-economic analysis. Technology with low energy conversion efficiency is unlikely to translate into a commercial application as it will suffer poor economic margins. It is also ethically important to maximize the utilization of low-carbon energy and avoid its conversion to waste heat.

**Figure 1:**
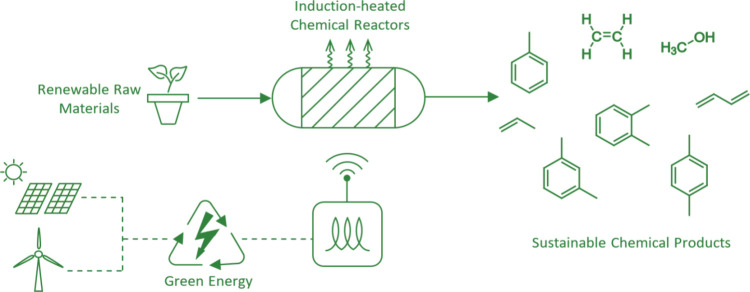
Illustration showing the key steps in harnessing renewable electricity to produce important chemical intermediates from renewable feedstocks by RF induction heating.

Example reactions for which green electrification would be a good option include ethylene production from bioethanol, syngas production from carbon dioxide by dry methane reforming; conversion of alcohols to liquid fuels; and biomass processing by gasification (Figure 1).^4^

The energy required for chemical conversions is conventionally delivered by thermocatalysis. Newer concepts such as electrocatalytic and photocatalytic conversion are promising alternatives although not yet at the same level of technology readiness. The heat for thermocatalytic conversion can be delivered by electrical resistance heating, radiofrequency (RF) induction heating, or microwave heating. An electrical resistance heater (Joule heating^5^) can achieve 100% energy efficiency but suffers from high heat flux which produces high surface temperature causing side reactions and fouling in some applications. Heat flux can be reduced by direct heating of the reactor wall by electrical resistance, although scale-up would likely require multiple reactor tubes which could be a practical problem.^6^ While useful at smaller scales, microwave reactors suffer from poor penetration depth^7^ and localized hot and cold regions when the wavelength is small compared to the reactor radius. This limits microwave reactors to the low centimeter scale, far below the requirement for large scale chemicals manufacture. Low temperature plasma catalysis is increasingly considered as a candidate for green routes to chemicals manufacture and this technique requires much lower catalyst temperature compared to conventional thermocatalytic routes. However, scale-up efficiency has recently been predicted to be around 19% and so further development is required for this technology to be deployed at scale.^8^

RF induction heating uses an external work coil to generate an alternating magnetic field, typically in the range of 1 kHz to 3 MHz. Magnetic or electrically conductive particles generate heat through magnetic hysteresis or eddy current formation. RF heating uniformly heats the whole susceptor bed and thus suffers no heat flux limitations or high surface temperatures. Induction heating for catalysis is a growing research field with a number of important recent developments. The heat generating particles within catalysts become effective hot spots and heat is produced in intimate proximity to the reaction sites, resulting in lower fouling, higher selectivity and less heat loss to the ambient environment.^9,10^ Further advantages include rapid ON/OFF control of reactors,^11^ and tailored axial thermal gradients which can boost conversion, rate and selectivity.^12^ Applications include organic synthesis reactions,^13^ water electrolysis^14^ and high temperature gas reforming catalysis.^15^ Bimetallic nanoparticles such as cobalt nickel and iron carbide have been demonstrated to heat rapidly and to high temperature.^16,15^

Two important barriers prevent the commercial development of RF induction heating. At a lab scale, typical measured energy efficiencies are in the range of 0.7–22%^16−18^ (efficiencies are variously defined as power to catalyst heat or to stored chemical energy) and the energy efficiency at a commercial scale has not been reported. Based on this lab scale data, it has been projected that induction heated catalytic reactors could deliver energy efficiency in excess of 80%: however in one case electromagnetic power losses were not quantified;^17^ and in another the power losses arising from the proximity effect were included, but losses from the skin effect were not.^18^ Furthermore, the impact on changing electromagnetic field strength and frequency as a result of increasing the work coil diameter for scale-up were not considered. It is therefore not clear whether these projections for energy efficiency are realistic when the full picture of power losses and scale-up aspects are included.

The induction work-coil is conventionally located outside of the reactor and thus the preferred electrically conductive steels cannot be used in the reactor construction with glass or ceramic being used instead. A recent proposal to locate the work-coil inside the reactor would allow the reactor wall to be made from steel.^19^ It would also allow the reactor wall to provide some electromagnetic shielding, an important safety and regulatory consideration. While this would solve two important problems, it is not clear how the work coil will perform at elevated temperature within the reactor vessel or whether the reactor wall, which is now external to the work coil, would generate excessive eddy currents impacting on the overall efficiency as it removes power from the field.

There is a clear need for a better understanding of the energy efficiency of RF induction heating at all scales of applicability. Previous studies have modeled losses arising from AC resistance in the induction work coil including the proximity effect and heat losses to the ambient environment.^18^ In this paper, a new comprehensive model of the energy losses is presented. It is valid for both hysteresis and eddy current heating and it gives the first overall picture of energy efficiency. The model is then applied to a case study continuous flow ethanol dehydration reaction so that the energy conversion efficiency for a real application can be seen.

## 2 Methods

### 2.1 Development of an RF Induction Efficiency Model

#### 2.1.1 The Effects of Reactor Scale on RF Induction Efficiency

**Figure 2:**
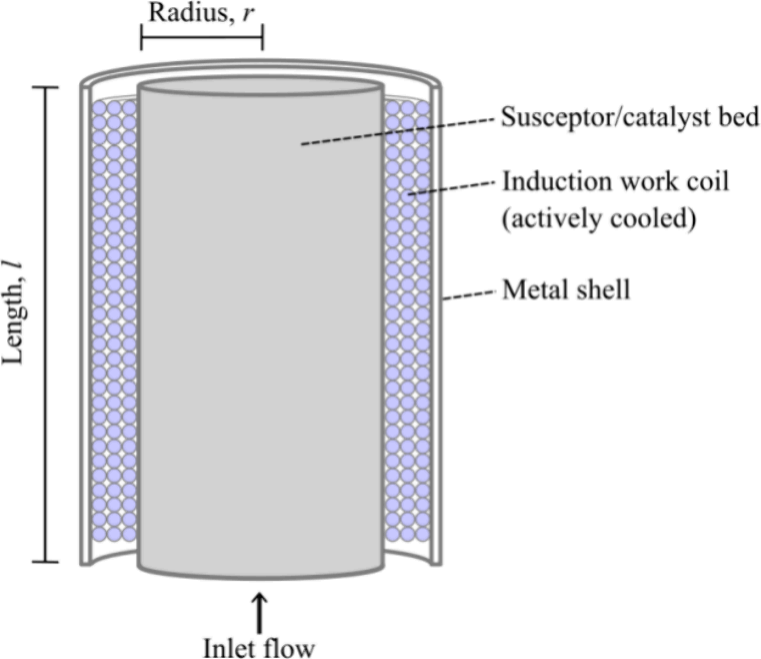
General arrangement of an induction-heated bed, showing a central bed of susceptor material inside an induction work coil with an outer pressure containment vessel made from metal. The ends of the vessel are not shown. The work coils are electrically insulated from one another, from the vessel wall and from the catalyst bed. The coils are in thermal equilibrium with each other and the bed.

Figure 2 shows an induction heated reactor with the work-coil located externally to the susceptor bed and inside of the vessel shell. Reactor dimensions are given in terms of the aspect ratio, *a*, of the reactor length to the diameter, and the number of coil turns per unit length of the work coil, *n*. This allow reactors of varying scale to be examined on an equivalent basis.

Induction heated reactors should have an approximately constant field strength. This follows from the quasi-static approximation and holds if the diameter of the reactor is much smaller than the wavelength, λ, associated with the operating frequency, *f.*(20) The wavelength and frequency are related through the speed of light and this sets an upper limit for the product of frequency and reactor radius (eq 1). Most industrial-scale reactors have a radius smaller than three metres, setting an upper operating frequency of 500 kHz for the constant field strength assumption to hold at the largest reactor scale.
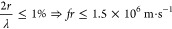
1

#### 2.1.2 Development of an Efficiency Parameter

There are two heating mechanisms for RF induction heating. Eddy currents are induced in electrically conductive materials. They act to oppose the externally applied magnetic field and heat is generated by the resistivity of the material. Magnetic hysteresis occurs when the internal magnetic dipoles of ferro- and ferri-magnetic materials align with the externally applied field and heat is generated by resistance to the change in their orientation, such as through crystalline anisotropy.

The overall heating efficiency, η, is defined in terms of an efficiency parameter, *p,* which is the sum of the power loss terms divided by the useful heat (eq 3). They are expressed on a per unit volume of reactor basis and are given as a function of the peak applied field strength.
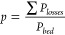
2
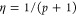
3In order to estimate the overall efficiency, expressions for the various power loss mechanisms and the useful heating power generated by magnetic hysteresis and eddy currents must be developed.

#### 2.1.3 Sources of Inefficiency: Power Losses

##### Power Supply Switching Losses and Ambient Heat Loss

**Figure 3:**
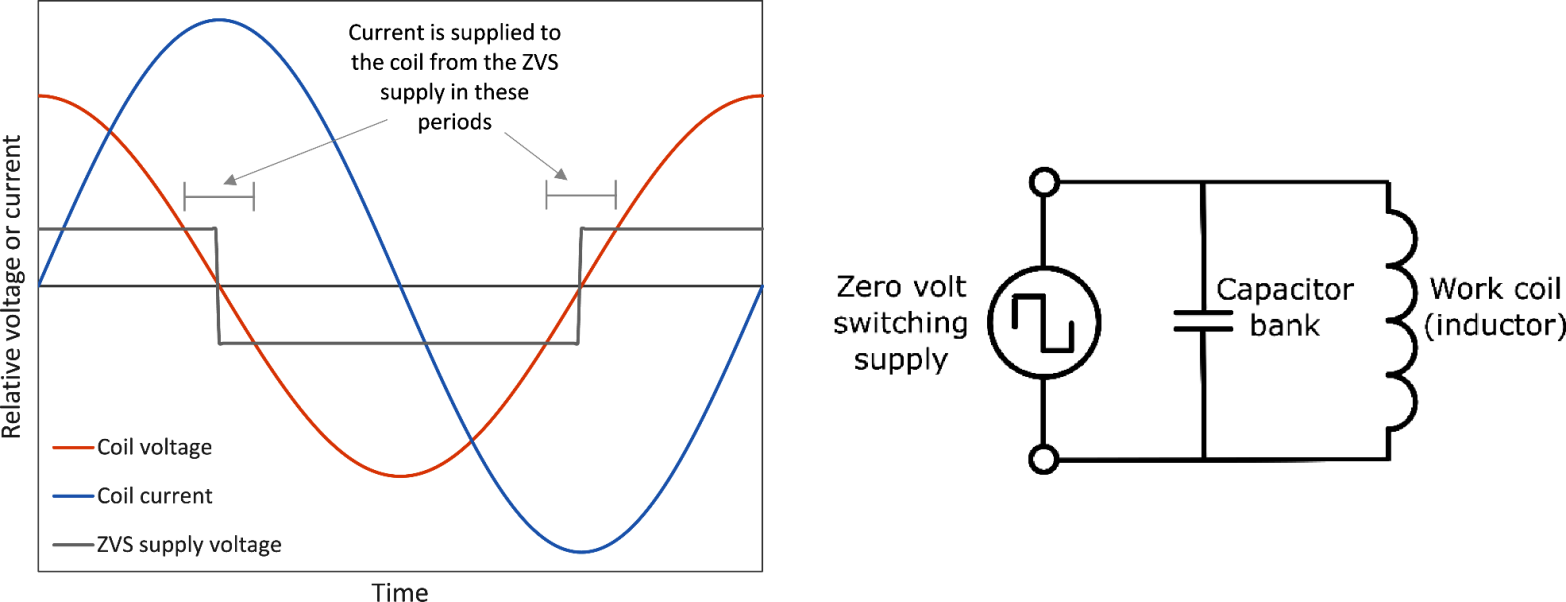
Simplified schematic of a resonant tank circuit showing the capacity bank, work coil and zero volt switching power supply. The chart shows an example voltage and current flowing through the work coil, and the voltage of the zero volt switching (ZVS) power supply, which provides current to the coil when the coil voltage is smaller than the supply voltage.

Electrical power for RF induction heating is supplied by resonant tank circuits in which a low frequency, low voltage supply is boosted to high voltage DC and then switched at high frequency using power transistors (see Figure 3). They can achieve one megawatt of heating power at 500 kHz with efficiency between 83 and 95%..^18,21,22^ The supply circuitry typically has very high efficiency and so switching losses are neglected in this work. It is assumed that sufficient external insulation can be installed so that heat losses to ambient are negligible.

##### Losses Due to Heating of the Vessel Wall

The metallic steel walls of the reactor will generate heat from induced eddy currents. In this study the metal shell is placed in close proximity to the induction coil in order to represent a worst-case loss. It is assumed that the vessel shell is nonmagnetic with a relative permeability of one and for a well-insulated vessel, the wall is equithermal with the reactor bed.

The applied field strength immediately outside the radius of a long solenoidal coil (*Ĥ*_*ext*_) can be related to the internal applied field strength (*Ĥ*) as a function of the aspect ratio, *a* (eq 4).^23^
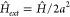
4Treating the reactor wall as a hollow cylinder, the power dissipated by induced eddy currents can be expressed as a function of *γ*_*v*_, the ratio of reactor radius to a parameter known as the skin depth, *δ*_*v*_ (eq 6). This is the solution to Maxwell’s electromagnetic equations in a cylindrical geometry, a combination of Kelvin functions, which are zero order modified Bessel function of the second kind, *K*_*0,*_ with an argument of *xe*^*i*. π/4^.^24^ This loss function is combined into a single vessel skin factor, *F*_*vessel*_ (eq 7) where *μ*_*0*_ is the permeability of free space, *σ*_*v*_ is the electrical conductivity of the vessel wall, and *F*_*vessel*_ is the vessel skin factor.
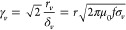
5
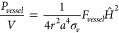
6
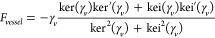
7
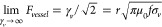
8At frequencies greater than 1 kHz, eq 8 approximates the vessel skin factor for electrically conductive vessels with a radius greater than 10 cm operating below 500 °C. This approximation applies to smaller radiuses as the electrical conductivity of the vessel and the operating frequency increases. eq 9 represents the power lost to wall heating for all induction heated reactors with the coil inside a metal shell. It is presented as an inequality as the power lost will be reduced if the metal shell is moved further away from the coil. The upper limit will be used throughout the rest of this work as it represents the most space-efficient method of packing a work coil into a metal reactor jacket. It shows that the vessel wall power losses per unit volume of reactor are inversely proportional to the radius of the bed, and rapidly fall as the aspect ratio increases
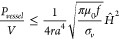
9

##### Work Coil Losses

A description of the power loss mechanisms within a work coil are described in this section. Additional information around the derivation of these equations are provided in the SI.

**Figure 4:**
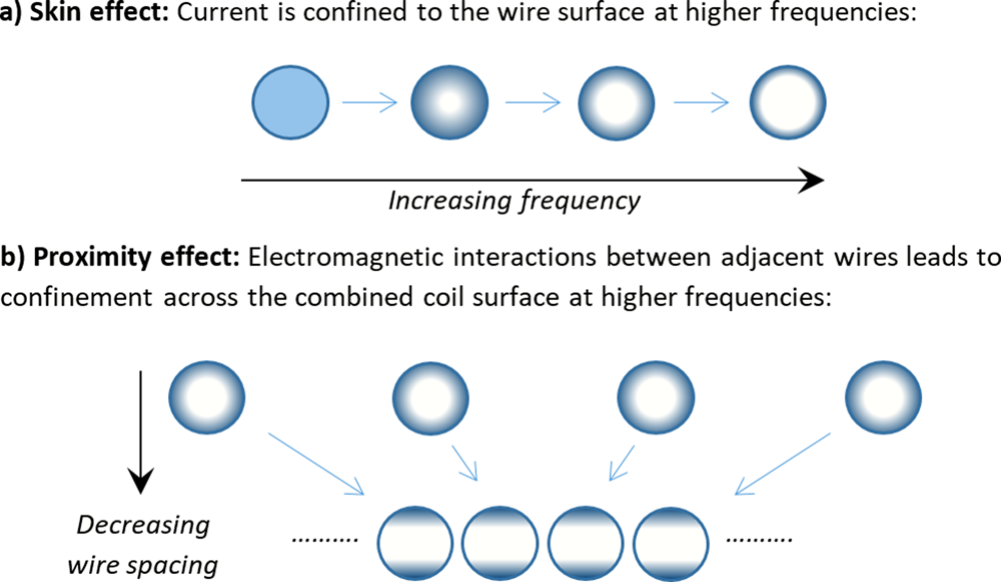
Illustration showing the skin and proximity effects on the current density within coil wires for AC current. In the skin effect, increasing the frequency results in the current being confined toward the outer surface of the wires. In the proximity effect, electromagnetic interactions between adjacent wires causes a reduction in area for flowing current and an increased effective resistance of the wire/coil assembly.

Electrical resistance losses in the work coil are magnified by the skin effect in which AC current density falls exponentially from the surface of the wire toward its center. This reduces the effective cross-sectional area and increases the overall resistance.^24^ At higher frequencies, hollow tubes have equivalent resistance to a solid wire. When coil windings are in close proximity, the current flowing in each is subject to electromagnetic interactions which further increase the apparent resistance. This is the proximity effect, and is illustrated in Figure 4.

The effective AC resistance of a wire is expressed by applying skin and proximity effect factors to the DC resistance, *R*_*DC*_ (eq 10) where *l*_*w*_ is the total length of the work coil circuit, *σ*_*w*_ is the electrical conductivity and *r* is the wire radius. The skin effect factor, *F*_*skin*_, and proximity effect factor, *F*_*prox*_, can be separated into two independent terms because the respective fields generated by current flowing in a wire and by current flowing in adjacent wires occur in planes at right angles to each other.^25^
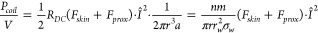
10

##### Skin Effect Parameter, F_skin_

**Figure 5:**
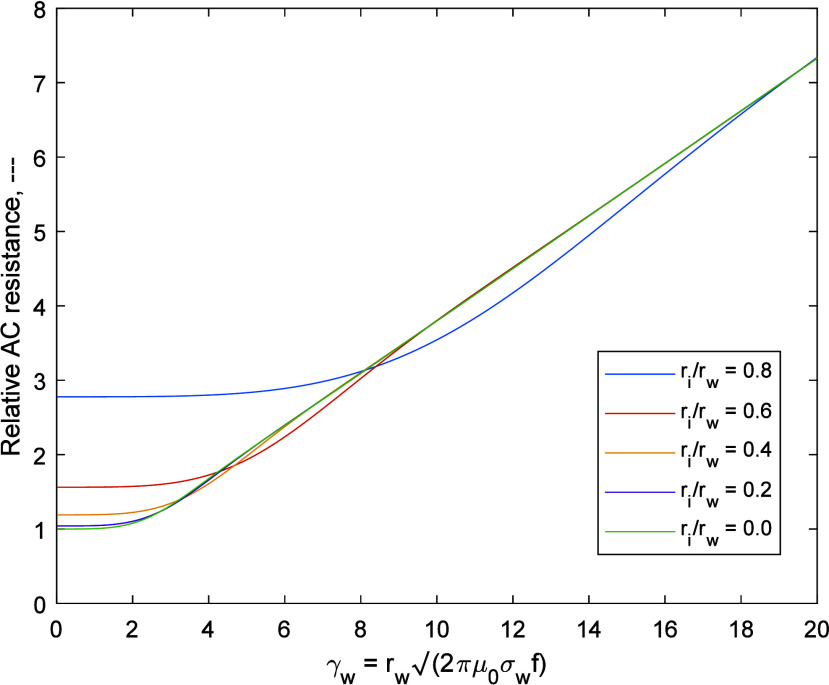
Relative AC resistance due to the skin effect for a solid wire (r_i_/r_w_ = 0) and hollow tubes with the same outer radius, as a function of γ_w_. The AC resistance at low γ_w_ is approximately the same as the DC resistance. At higher frequencies, the resistance of the tubes converge on the resistance of the solid wire, as the current is forced toward the outer radius of each of the conductors.

The depth that a flowing alternating current penetrates into a wire or hollow tube is a function of frequency and wire electrical conductivity, σ. The increase in effective resistance of a wire or hollow tube due to the skin effect, *F*_*skin*_, has an analytical solution from Maxwell’s electromagnetic equations. It is a function of the wire or tube outer radius, *r*_*w*_ and optionally the inner radius of the tube, *r*_*i*_, in the case of hollow tubes. The skin depth parameter, *γ*_*w*_, is the ratio of wire radius to skin depth scaled by a factor of . The work coil material is assumed to be nonmagnetic and so has a relative permeability equal to one. A parameter A* is introduced to account for the reduction in the conductor cross-sectional area for hollow tubes. In this work, the solid wire is considered as a special case of the hollow tube, in which the internal radius is equal to zero, and hence A* takes a value of 1 for solid wires.
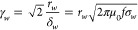
11
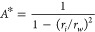
12
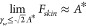
13
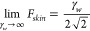
14At low frequencies, when the skin depth is larger than the wire diameter, current flows across the full conductor cross-section and the AC resistance is approximately equivalent to the DC resistance. The skin effect parameter, *F*_*skin*_, is an increasing function of *γ*_*w*_, and is directly proportional to it at very high frequencies. The resistance of a hollow tube at low frequencies also approximates to the DC resistance. At very high frequencies, the skin depth is small compared to the tube wall thickness and the AC resistance of the hollow tube converges on that of a solid wire with the same outer radius. The tube correction parameter and skin parameter of hollow tubes with different ratios of inner to outer diameter are compared in Figure 5.

**Figure 6:**
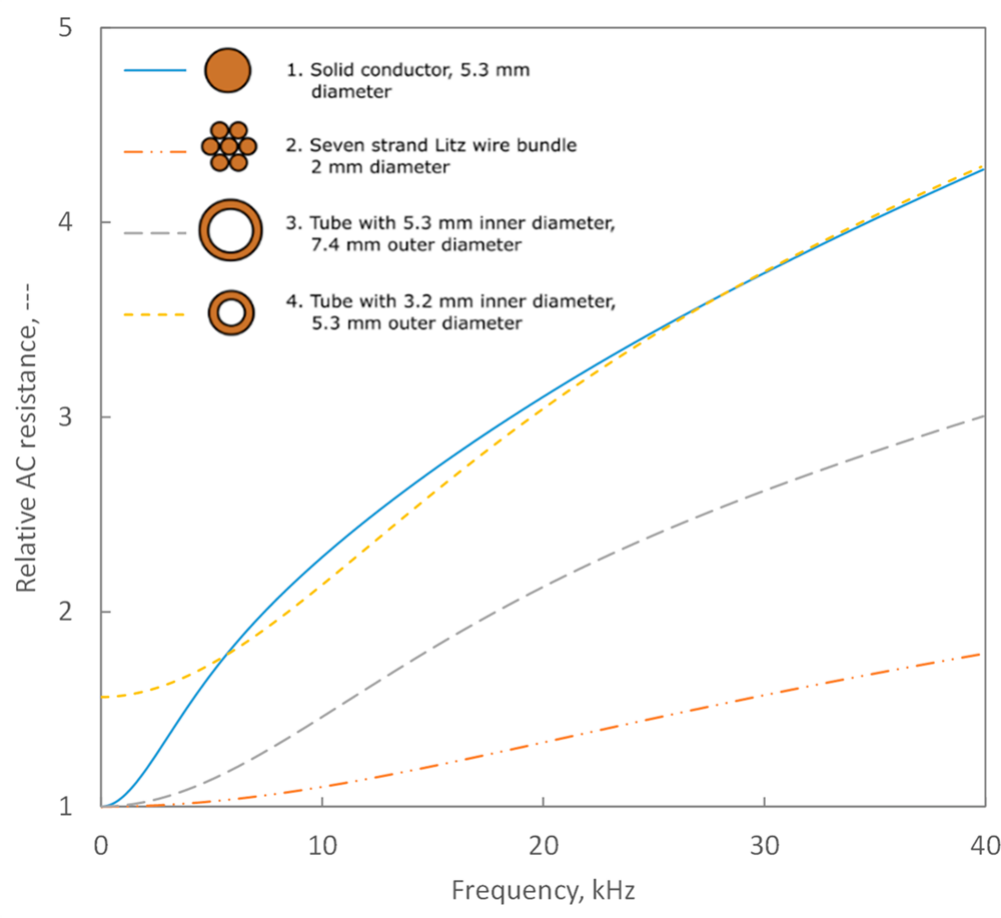
Skin effect as a function of wire geometry. Results for AC resistance versus frequency for identical cross-sectional area and DC resistance. (1) Single solid copper wire; (2) seven-strand copper Litz wire (no losses due to the proximity effect); (3) hollow copper tube; (4) hollow copper tube with the same diameter as the solid wire (this has a smaller cross-sectional area and higher relative DC resistance).

An illustration of the skin effect on the resistance of a solid copper wire, seven-stranded wire bundle and hollow tube with equal cross-sectional areas is given in Figure 6. The stranded wire is not subject to the proximity effect, which can be achieved with a special material called Litz wire. It has a lower resistance at higher frequencies than the solid wire as the ratio of strand radius to the skin depth in copper is smaller than in a single solid wire of larger diameter. The higher frequency resistance of the tube with the same area as the solid wire is smaller as the current fully penetrates the tube wall until the skin depth is smaller than the tube wall thickness. A fourth example consists of a hollow tube with the same outer diameter as the solid wire, and the same wall thickness as the previous hollow tube, 2.1 mm. This has a smaller area than the other three samples and a higher initial resistance. As the frequency increases the AC resistance converges on that of the solid wire with the same outer diameter.

##### Proximity Effect Factor, F_prox_

The proximity effect factor is a function of the ratio of the wire spacing to the wire diameter.^25,26^ This is equivalent to the diameter of the wire multiplied by the number of coil turns per metre. For a multiturn coil made from solid wire, the proximity factor is given by eq 15. The limits of the Bessel functions are given at low and high skin depth ratios in eq 16 and eq 17.
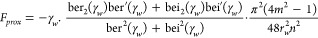
15
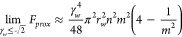
16
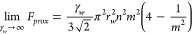
17The proximity effect for tubes is more complex as it must account for the tube’s hollow core. However, at high frequency, the proximity effect will be similar to that for a solid wire, as the skin effect restricts the current to the outer surface of both in the same manner. The results presented in Figure 6 demonstrates that the AC resistances of a copper wire and hollow tube of 5.3 mm radius are roughly equal above 5 kHz. Thus, it is assumed that the proximity effect for tubes can be approximated to the proximity effect in solid wires at high frequency.

#### 2.1.4 Relating the Coil Current to the Applied Field Strength

**Figure 7:**
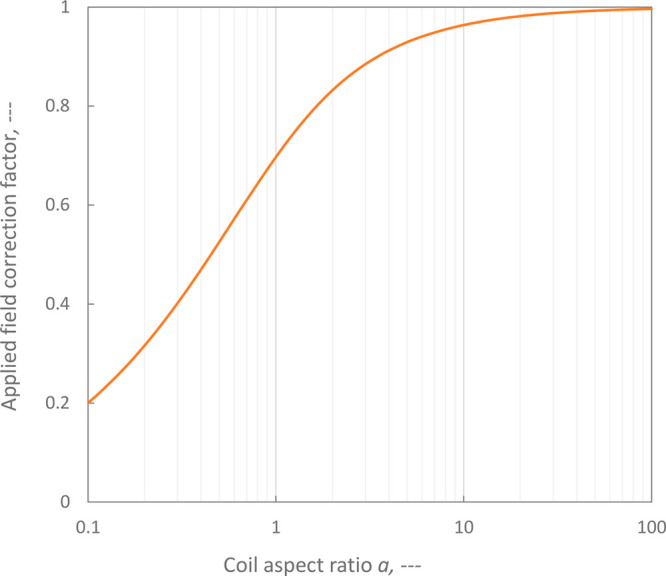
Applied field correction factor for a nonideal (short) solenoid, K, as a function of the ratio of reactor length to diameter (aspect ratio, a, eq 19) derived from Wheeler (1982).^27^

The magnetic field inside an ideal solenoidal coil can be derived from the Biot-Savart law, with the applied field strength dependent on the work coil current and geometry. In a nonideal coil, end effects reduce both the axial and radial field strength. This can be incorporated by applying a correction factor, K, to the Biot-Savart law (eq 18). In this work, the authors adapt Wheeler’s formula^27^ to be a function of coil aspect ratio to provide a simple relationship for the applied field correction factor, *K*, with a maximum relative error of 1.7%.
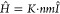
18
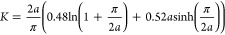
19The applied field correction factor, *K,* is an increasing function of the aspect ratio, exceeding 0.7 at an aspect ratio of one and asymptotically approaching unity for higher aspect ratios (see Figure 7).

#### 2.1.5 The Total Power Losses

The expression for the magnetic field within the coil (see SI) and eq 19 relate the work coil current to the applied field strength and enable an expression for the total power losses per unit volume (eq 20) that includes heating of the vessel walls and work coil losses by the skin and proximity effects. Increasing the aspect ratio reduces the power losses per unit volume for all loss terms. As the aspect ratio increases, the applied field strength external to the coil falls, end losses become less significant and the field strength inside the coil is greater for a given coil current (Figure 7).
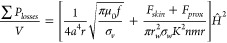
20

## 3 Results

### 3.1 Induction Heating Power of a Reactor Bed

#### 3.1.1 Heating Power of a Magnetic Hysteresis Bed

**Figure 8:**
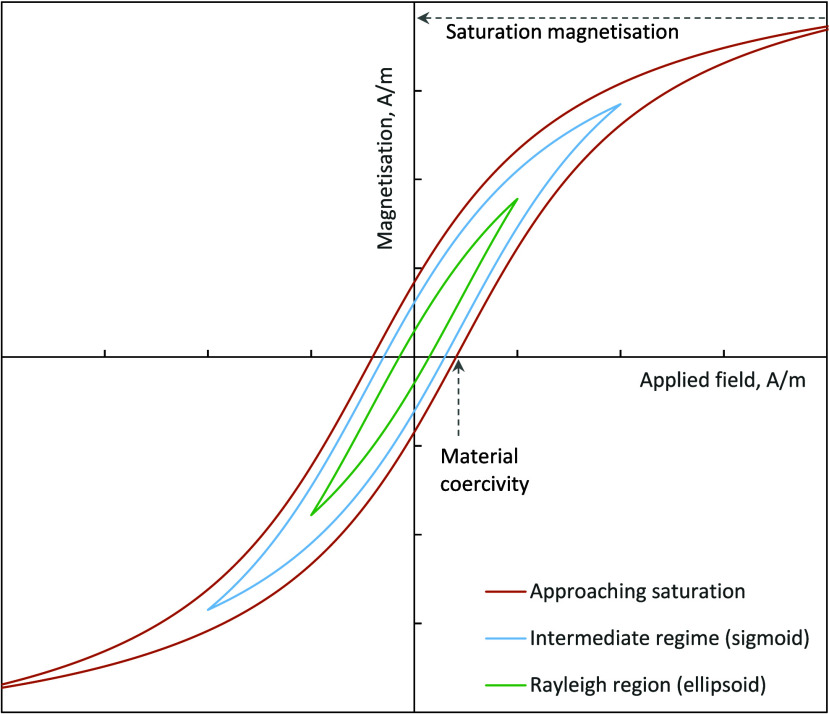
Example magnetization curves for a soft magnetic material, such as magnetite, showing: the characteristic ellipsoidal curve denoting the Rayleigh magnetization region, where the applied field is less than 250% of the material coercivity; an intermediate regime where the hysteresis curve becomes sigmoidal; and the approach to saturation with an approximately full hysteresis loop. Each of these regions is characterized by a different heating power as a function of increasing applied field strength.

Example hysteresis loops showing the Rayleigh, intermediate and approach to saturation magnetization curves are given in Figure 8 where *H* is the applied field strength, *P*_*hys*_ is the hysteresis heating power (proportional to the loop area), υ is the Rayleigh parameter and *χ*_*r*_ is the Rayleigh law tip susceptibility. The maximum specific heating power of maghemite and magnetite nanoparticles occurs just beyond the Rayleigh region,^28^ in the limit that the peak applied field is less than 250% of the major hysteresis loop coercivity, *H*_*c*_. The unit volume heating power in the Rayleigh region can be derived from Rayleigh’s law (eq 21).^29^
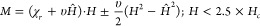
21
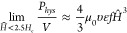
22

The Rayleigh parameter is material specific and a function of temperature. It is assumed to be a constant bed-averaged property. At very high applied field strengths the magnetic material becomes completely saturated and the hysteresis loop area tends toward a fixed value. In these circumstances, the hysteresis loop area, and hence power, is proportional to the product of saturation magnetization, *M*_*S*_, and major loop coercivity, *H*_*C*_.^30^
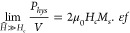
23The heating power of a sample in the intermediate region between the Rayleigh region and saturation can be determined using a semitheoretical arctangent model that approximates the major and minor hysteresis loops and power for magnetite and maghemite soft ferrites as a function of *y*_*0*_, *A*, *x*_c_ and *w* (eq 24 and eq 25).^28^ The parameter *A* is analogous to the saturation magnetization, *x*_*c*_ is analogous to the coercivity and *w* is the full width at half-maximum (fwhm) of a Lorentzian distribution fitted to the hysteresis loop susceptibility. This allows calculation of the energy efficiency for heating of a known magnetic material and simplifies to the Rayleigh law (eq 22) and saturation power case (eq 23) at low and high values of applied field, respectively.^28^
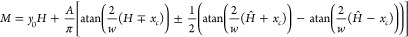
24
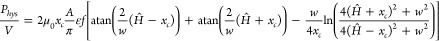
25The Rayleigh regime (eq 22), intermediate sigmoidal region (eq 25) and approach to saturation (eq 23) can be expressed as a single hysteresis factor, *F*_*hys*,_ which takes a value of 4*υĤ*/3 in the Rayleigh region and 2*H*_*c*_*M*_*s*_/*Ĥ*^2^ in the approach to saturation.
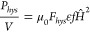
26The useful hysteresis heating power is combined with the total power losses in the work coil and vessel wall to give a general equation for the hysteresis efficiency parameter, *p*_*hys*_, (eq 27) with results presented in Table 1.

**Table 1:** Summary of the Terms Influencing the Efficiency Parameter for the Generalized Hysteresis Heating of Magnetic Materials, *P*_Hys_^a^

Limits	Efficiency Parameter, *P*_*Hys*_	
General case	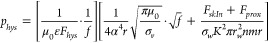	*Eq 27*
*Ĥ* < 2.5*H*_*C*_	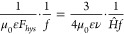	*Eq 28*
*Ĥ* ≫ *H*_*C*_	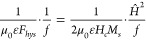	*Eq 29*
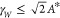	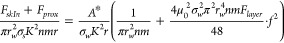	*Eq 30*
γ_*W*_ → ∞	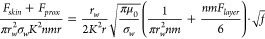	*Eq 31*

a The hysteresis heating factor F_hys_ is a function of applied field strength, and the parameters F_skin_ and F_proximity_ are functions of the skin depth and hence frequency. The coil layer factor, F_layer_, is equal to 12π for a single layer coil and approximates 16π for coils with two or more layers (see SI for further details). The efficiency of the system is maximised by minimising p_hys_ (eq 3).

#### 3.1.2 Induction Eddy Current Heating Power of a Bed of Conductive Spheres

Radio-frequency induction heating of electrically conductive materials, such as a bed of conductive spheres. They must not be in contact with each other to prevent formation of interparticle and circulating eddy currents which would be significantly greater at the outer radius of the bed Only nonmagnetic susceptor materials are considered due to the complex interaction between eddy current and hysteresis heating mechanisms.^31^

**Figure 9:**
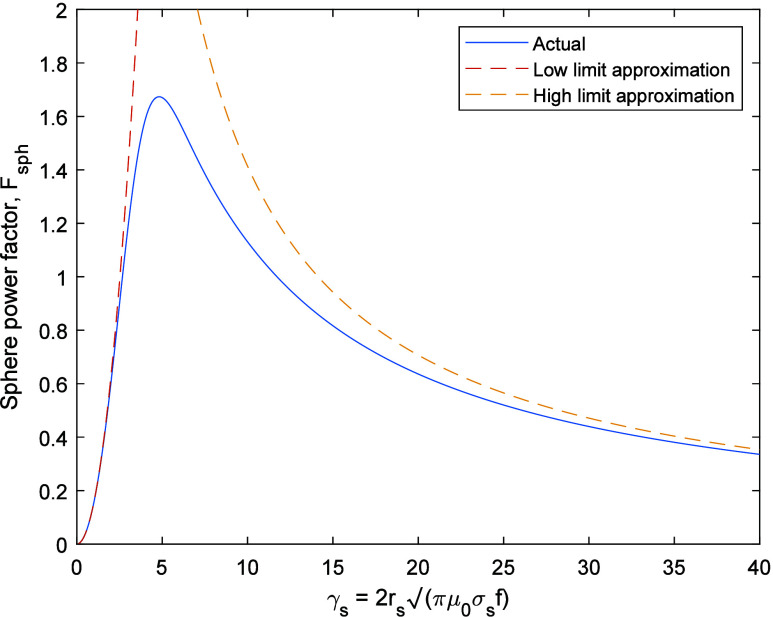
Eddy current heating sphere power factor, F_sph_ as a function of the ratio of sphere diameter to skin depth in the sphere, γ_s_ (eq 34) along with the low limit approximation (γ_s_ < 2, eq 35) and high limit approximation (γ_s_ → ∞, eq 36). The function peaks at a value of 1.67 for γ_s_ = 4.8, implying an optimum operating point for maximizing the heating power of spheres in an induction heating system design.

There is a direct analytical solution of Maxwell’s equations for the eddy current power of a single, electrically conductive, nonmagnetic sphere in a uniform magnetic field^32,33^ and this gives the heating power per volume (eq 33). The volume fraction, ε, takes an upper limit of 74% (densely packed spheres).^34^ A sphere power factor, *F*_*sph*_, is defined as a function of frequency, material properties and sphere radius (eq 34), and this can be simplified when the skin depth is large compared to the sphere radius (eq 35) and when it is very small (eq 36). Figure 9 shows that it reaches a maximum when the sphere diameter to skin depth ratio, *γ*_*s,*_, is 4.8.
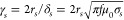
32
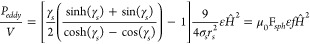
33
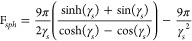
34
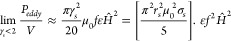
35
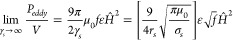
36Where *P*_*eddy*_ is the eddy current heating power of a sphere, *r*_*s*_ is the spherical heating particle radius, *σ*_*s*_ is the sphere electrical conductivity, *F*_*sph*_ is the sphere power factor *and δ*_*s*_ is the sphere skin depth.

The efficiency parameter for eddy current heating of spheres is presented in Table 2, The eddy current efficiency is maximized by minimizing the efficiency parameter.

**Table 2:** Summary of the Equations Defining the Efficiency Parameter for the Generalized Eddy Current Heating of Spheres, p_eddy_^a^

Limits	Efficiency Parameter, *p*_*eddy*_	
General case	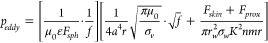	*eq 37*
*γ*_*s*_ ≤ 2	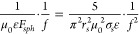	*eq 38*
*γ*_*s*_ → ∞	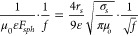	*eq 39*

a The work coil loss parameters F_skin_ and F_proximity_ are functions of the skin depth and frequency, as is the sphere power factor. The vessel wall and work coil loss term is identical to the hysteresis case and are given in eqs 30 and 31. The coil factor, F_layer_, is equal to 12π for a single layer coil and approximates 16π for coils with two or more layers (see SI).

#### 3.1.3 Summary of Parameter Influence on the Energy Efficiency of Reactor Heating

The variables that impact the efficiency of eddy current and hysteresis heating are given in Table 3. The efficiency continuously increases as the reactor radius and volume fraction of material in the reactor increases. Higher aspect ratios are also advantageous for high efficiency, either explicitly in the vessel wall loss or implied in the work coil loss through the presence of the applied field correction factor term, which is an increasing function of aspect ratio. Above a certain aspect ratio, the work coil loss terms become dominant over the vessel wall losses, and at higher ratios the applied field correction factor asymptotically approaches a value of one.

**Table 3:** Effect of Various Parameters on the Efficiency of Induction Heated Reactors That Follow from the Equations Developed in This Work^a^

	Parameter	Effect on Energy Efficiency
**Operating parameters**	Frequency, *f*	Hysteresis energy efficiency increases with frequency when the skin depth in the coil is much smaller than the wire thickness. At low frequency the coil proximity factor dominates. For eddy current heating, efficiency increases from low to medium frequency when the skin depth is small in both the coil wires and the spheres.
	Applied field strength, *Ĥ*	Hysteresis efficiency is maximized when the applied field strength passes the major loop coercivity and then falls at higher field strength. Eddy current heating efficiency is independent of field strength.
**Reactor geometry and material**	Radius, *r*	Efficiency increases as a function of bed radius.
	Aspect ratio, *a*	Efficiency increases as a function of aspect ratio until the coil approximates an ideal solenoid.
	Volume fraction of heating material, ε	Efficiency increases continuously.
	Vessel electrical conductivity, *σ*_*v*_	Efficiency increases continuously with higher vessel electrical conductivity.
**Coil parameters**	Wire electrical conductivity, *σ*_*w*_	Efficiency increases with increasing wire conductivity at high frequency. The effect at low frequency depends on the coil proximity factor.
	Number of turns per metre, *n*	The coil design has a complex effect on efficiency and is a function of DC resistance, skin effect and proximity factor.
	Number of coil layers, *m*	
	Wire radius, *r*_*w*_	
**Hysteresis Susceptor Properties**	Material coercivity, *H*_*c*_	Efficiency increases with susceptor coercivity.
	Rayleigh parameter, ν	Efficiency increases with Rayleigh parameter when the applied field is below the Rayleigh limit
	Saturation magnetization, *M*_*s*_	Efficiency increases with saturation magnetization if the applied field is above the Rayleigh limit.
**Eddy Current Susceptor Properties**	Sphere radius, *r*_*s*_	Efficiency initially increases with increasing sphere radius or conductivity and then decreases as the skin depth becomes smaller than the sphere diameter.
	Sphere electrical conductivity, *σ*_*s*_	

a Increasing efficiency requires decreasing values of efficiency parameters (p_hys_ or p_eddy_). Note that this does not include the efficiency of chemical conversion which varies and is specific to a particular reaction.

The efficiency of eddy current heating is independent of the applied field strength. Increasing the applied field strength in hysteresis heating has an initial positive effect on the efficiency, reaching a peak when the applied field passes the susceptor coercivity then rapidly falls as it approaches saturation. At low frequency, the frequency effect is complex and depends on the balance between the skin and proximity effects in the coil. At higher frequencies, in which the skin depth in the coil is small, the efficiency continues to increase for hysteresis heating and becomes constant for eddy current heating.

The susceptor material selection for magnetic hysteresis is important. A larger coercivity allows for operation at a larger applied field strength and this increases efficiency. A larger Rayleigh parameter leads to a higher heating power per unit volume and a higher efficiency, whereas higher saturation magnetization appears to be advantageous only if the reactor operates in a region where the material is approaching saturation. The Rayleigh parameter and saturation magnetization are not independent as they are linked through the intrinsic magnetic properties of the material. For eddy current heating, the sphere radius and electrical conductivity can be optimized to produce a peak in efficiency as shown in Figure 9.

### 3.2 Case-Study Application of an Induction Heated Flow Reactor for Ethanol to Ethylene

**Figure 10:**
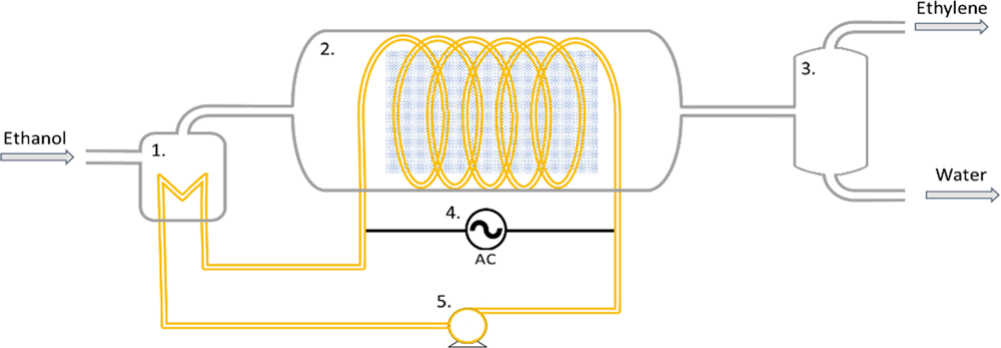
Example process flow schematic for the ethanol dehydration reaction showing potential for heat integration and product separation. 1 - ethanol feed heater and vaporiser; 2 - induction heated catalytic reactor with magnetic susceptor catalyst bed and work coil located within the reactor vessel; 3 - product separator; 4 - high frequency RF generator resonant tank circuit; and 5 - circulating heat transfer fluid.

The equations determining the energy efficiency for reactor heating are now applied to a model ethanol dehydration flow reaction heated by either induced magnetic hysteresis or eddy currents. The reaction is endothermic and produces ethylene, a building block chemical whose normal production is highly carbon intensive. Gas-phase ethanol is dehydrated to ethylene over ZSM-5 at 225 °C (+44.9 kJ·mol^–1^)^35^ which can be easily accomplished using induction heating.^28,36^ The heating material and catalyst are combined together in a pellet form with 50% void fraction. The required heating power is 490 kW·m^–3^. An illustration process flow schematic with internal work coil and work coil heat integration is shown in Figure 10. The continuous plug flow reactor design is likely to be advantageous for induction heating at scale as it both minimizes and fixes the catalyst bed volume while allowing large throughputs.

#### 3.2.1 Coil Heat Dissipation Requirements

At steady state, the heat generated is equal to the heat removal from the coil given by the maximum heat flux across the coil’s surface. A conservative heat transfer coefficient of 400 W·m^–2^·K^–1^ is used and a temperature difference between the coil and cooling fluid is taken to be 5 °C to give a heat flux across the coil surface of 2000 W·m^–2^.^37^ The limiting heat flux, *p*_*flux*_, is given as a function of *r*_*x*_, a characteristic wire radius, defined as *r*_*w*_ for a solid wire of the outer surface of a tube, *r*_*i*_ for the inside surface of a tube, and *(r*_*i*_*+r*_*w*_*)* for cooling applied to the inside and outside surfaces of a hollow tube.
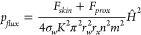
40In setting a limit on the heat flux, eq 40 sets a dependent relationship between the applied field strength and the skin effect and proximity effect factors, which are functions of the operating frequency (See SI and eq 15). The catalyst power requirements then set a fixed relationship between the frequency, field strength and heating material volume fraction (eq 26). This means that the required applied field strength and volume fraction of heating material are constrained by the operating frequency.

#### 3.2.2 The Effects of Resonant Tank Circuits

The derivation of the efficiency parameter assumed that frequency is an independent variable. It then followed that frequency, applied field strength and the required volume fraction of heating material were independent of the reactor scale. However, frequency in a resonant tank circuit is a function of the inductance of the work coil and susceptor bed which is a function of these parameters and so the efficiency model must be adapted to reflect the characteristics of the resonant tank circuit.

When the resonant tank circuit is at the resonant frequency, the energy stored in the magnetic field of the inductor at peak current and zero voltage is equal to the energy stored in the capacitor electric field at peak voltage and zero current (eq 41). The resonant frequency occurs when the combined circuit reactance is equal to zero (eq 42).
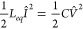
41
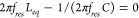
42
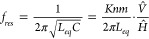
43The inductance of the empty work coil is proportional to the reactor volume. It increases with reactor scale and must be matched with an increase in capacitance or maximum voltage in order to satisfy the work coil circuit energy balance (eq 41). However, eq 43 shows that the capacitance must remain inversely proportional to the inductance in order to prevent the resonant frequency, and hence efficiency, from falling at larger reactor scales. It follows that maximum voltage across the work coil must increase to satisfy these criteria and to give the most efficient design.

The total inductance of the combined work coil and susceptor bed is determined by defining a relative permeability associated with the eddy current or magnetic material based on the volume fraction of heating material in the bed and an equivalent susceptibility, which is independent of the bed volume.
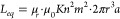
44
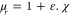
45
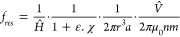
46The resonant frequency produced by a resonant tank circuit falls with the cube of the radius and this has a significant detrimental effect on efficiency of induction heated reactors at larger scales unless it can be offset by increases in voltage.

#### 3.2.3 Susceptibility of a Bed of Magnetic Material

The susceptibility for the magnetic hysteresis bed, *χ*_*hyst*_ is given by the ratio of maximum magnetization to the applied field strength. In the Rayleigh region, it is a function of *χ*_*r*_, υ and the applied field strength. It falls toward zero as the sample is saturated at high field strengths. The resonant frequency for magnetic hysteresis is inversely proportional to a power of the applied field strength between linear and squared. This causes a reduction in frequency that would reduce the efficiency as described in Table 3.
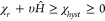
47

#### 3.2.4 Susceptibility of a Bed of Electrically Conductive Spheres

The eddy current circulating within a single electrically conductive sphere can be represented as an equivalent magnetic dipole moment, *m*_*sph*_, opposed to the applied field (eq 48).^32,38^ The equivalent eddy current susceptibility, *χ*_*eddy*_, is obtained by dividing this moment by both the peak applied field strength and the volume of the individual sphere. The eddy current susceptibility is proportional to the square of the resonant frequency at low frequencies, and approaches a constant value at high frequency.
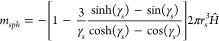
48
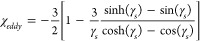
49
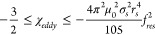
50The effect of applied field strength and scale on efficiency are less than the hysteretic case, especially at the lower resonant frequencies which would occur in the case of larger reactors.

### 3.3 Heating Efficiency for Ethanol Dehydration over a ZSM-5 Catalyst – An Example

The model RF induction reactor has a single layer work coil of tightly packed 1 mm radius copper wire (500 turns per meter), contained in a stainless steel vessel with an aspect ratio fixed at 2:1. The wire electrical insulation thickness is assumed to be negligible in relation to the wire radius. The susceptor material for magnetic hysteresis is 97 nm magnetite powder which has been characterized previously^28^ with hysteresis model parameters (eq 25) of *x*_*c*_ = 12 400 A·m^–1^, A = 121 100 A·m^–1^, w = 53 500 A·m^–1^and y_0_ = 0.8 at 225 °C. The susceptor material for the eddy current heating case is a bed of stainless steel balls of radius 5 mm coated with ZSM-5 catalyst. In both cases, the combined catalyst/susceptor pellets have a void fraction of 50% within the bed.

**Figure 11:**
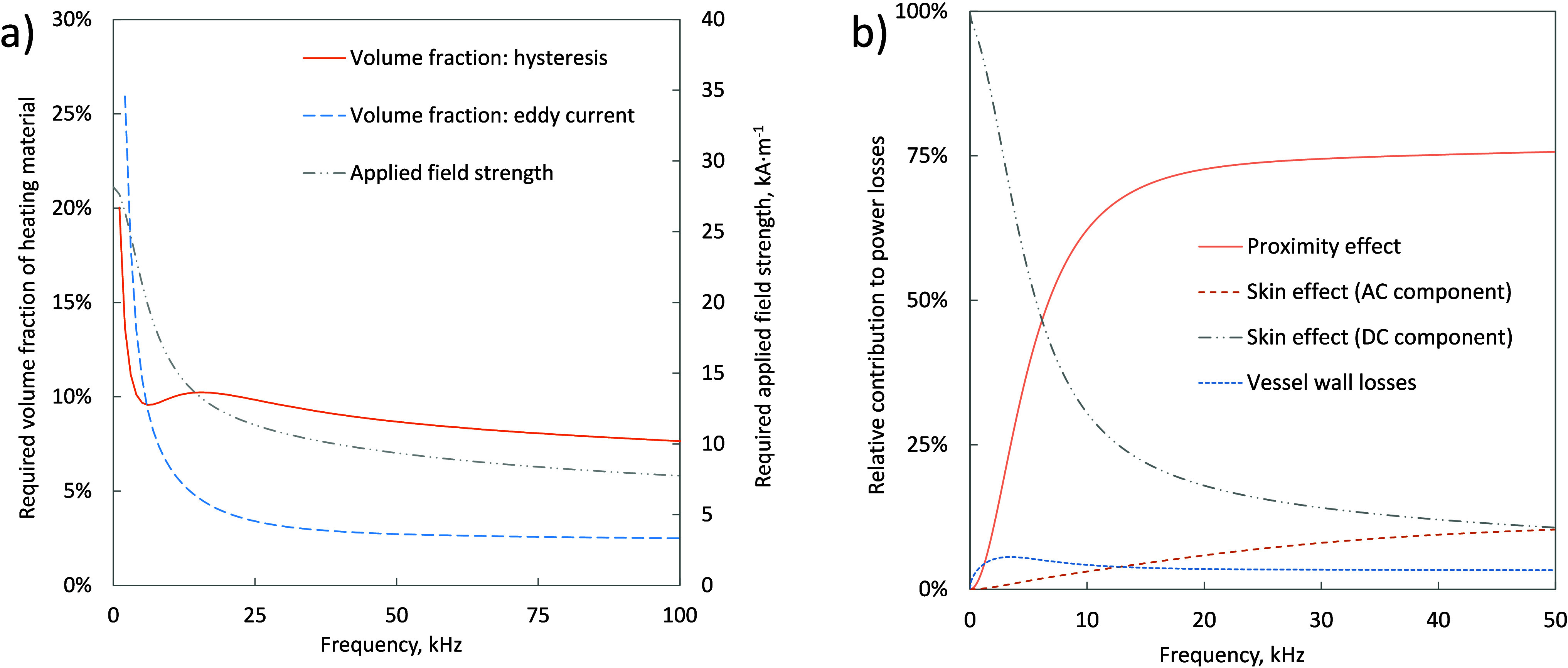
Energy efficiency for the ethanol to ethylene reaction over a ZSM-5 zeolite at 225 °C induction heated in a stainless steel vessel with an aspect ratio of 2:1 and heated by a single layer coil made from 1 mm radius copper wire with 500 turns/m, with a packed bed total void fraction of 50% and a limiting heat flux, p_flux_, of 2 kW·m^–1^. The susceptor materials for Eddy current and hysteresis heating are 5 mm radius stainless steel balls and 97 nm magnetite particles, respectively. (a) Required volume fraction of heating material, ε, in the reactor for eddy current and hysteresis heating, and the required applied field strength; (b) relative contribution of skin effect, proximity effect, and vessel wall losses to the total losses as a function of frequency. The relationships presented are independent of the heating material and reactor scale. The skin effect losses have been split out into those associated with the DC resistance of the work coil (F_skin_ = 1) and the excess resistance associated with operating the work coil at higher frequencies.

The required volume fraction of heating material required for eddy current and hysteresis heating is given in Figure 11a. In all cases the applied field strength and frequency are constrained by the heat flux limitation from the work coil (eq 40), and there is a one-to-one relationship between the operating frequency of the reactor and the required applied field strength (Figure 11a). The relative proportion of vessel wall losses, and losses from the coil resistance and excess resistance due to the skin proximity effects are solely a function of frequency, regardless of the susceptor heating material or radius of the reactor bed (Figure 11b) and this agrees with experimental results in literature.^18^

Figure 11 shows that the relative contribution of each loss term is approximately constant above 25 kHz, with a required applied field strength in the range of 8–11 kA·m^–1^. The contribution due to induction heating of the vessel wall is consistently less than 6% of the total. At low frequency, the losses are dominated by the skin effect term, which includes the DC resistance of the work coil. At higher frequencies, the proximity effect dominates the loss term. The results demonstrate that higher frequencies are not necessarily advantageous for induction heating and they may not be achievable with resonant tank circuits because they would require further increase in the work coil voltage above the present practical limit.

**Figure 12:**
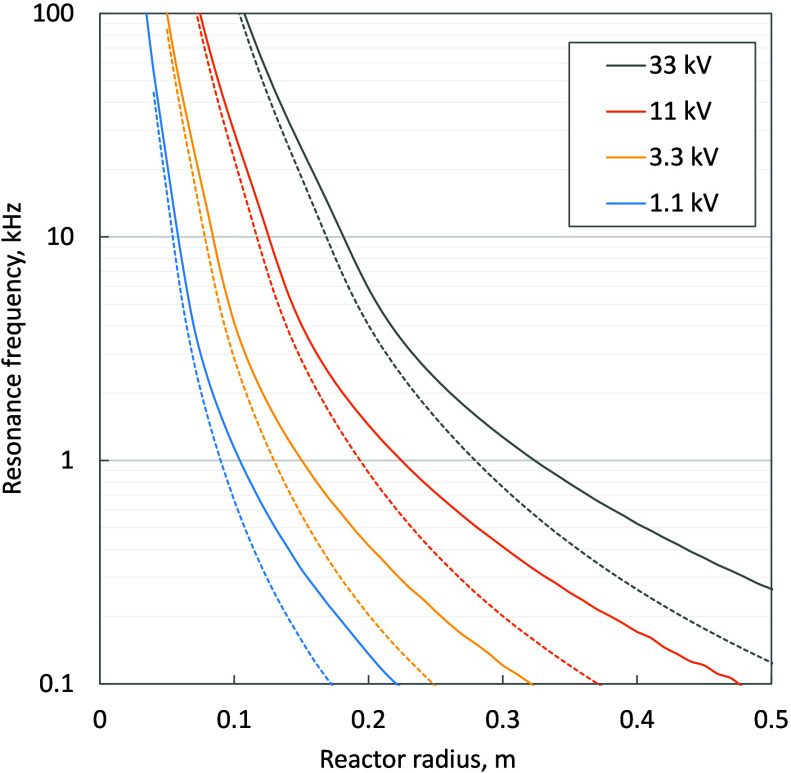
Resonant tank frequency as a function of reactor radius and peak voltage for eddy current heating (solid lines) and hysteresis heating (dashed lines) in the ethanol to ethylene reaction over a ZSM-5 zeolite at 225 °C induction heated in a stainless steel vessel with an aspect ratio of 2:1 and heated by a single layer coil made from 1 mm radius copper wire with 500 turns/m, with a packed bed total void fraction of 50% and a limiting heat flux, p_flux_, of 2 kW·m^–1^.

Figure 12 shows the effect of peak voltage and reactor size on the resonant frequency for eddy current and magnetic hysteresis heated reactors. It is practically difficult to achieve resonant frequencies in excess of 10 kHz with reactors larger than 15 cm radius.

**Figure 13:**
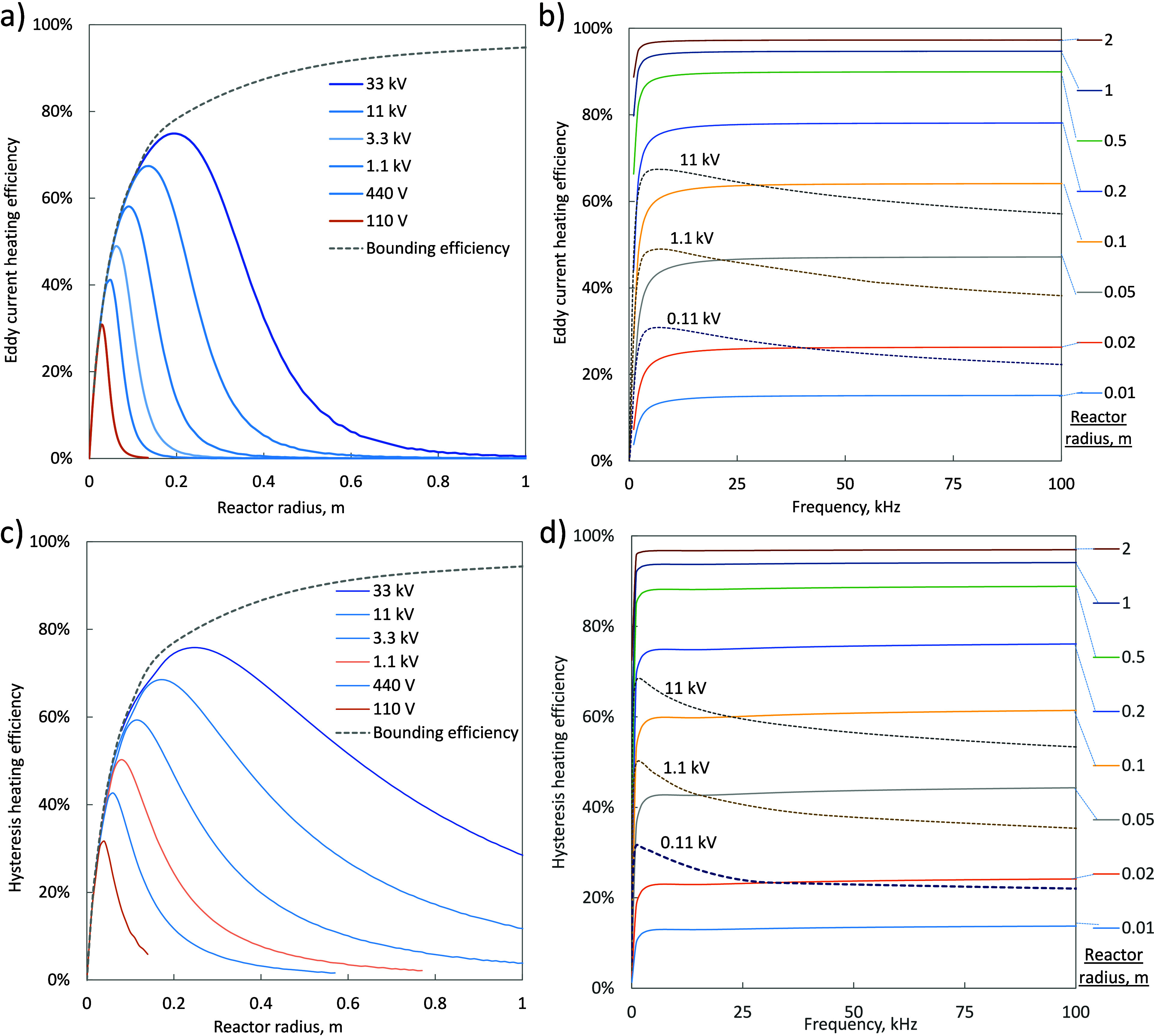
(a) Effect of the resonant frequency on the maximum achievable efficiency for eddy current heating of a given reactor radius at varying voltage levels. The bounding curve is the theoretical efficiency at a fixed frequency of 500 kHz; (b) efficiency for eddy current heating when the frequency is not constrained by a resonant tank circuit for varying reactor radiuses (solid lines) overlaid with curves for the resonant frequency at various voltage (dashed lines). The intersection of the solid and dashed line represents the operating point for a resonant tank circuit for that combination of voltage and radius; (c and d) magnetic hysteresis equivalent of curves (a) and (b) using magnetite powder as a heating medium.

Figure 13 shows the performance of a large scale induction heated reactor driven by a resonant tank circuit. If the frequency could be controlled as an independent variable, rather than being set by the resonant frequency, then the required volume fraction of heating material in the reactor is stable above 25 kHz, requiring the susceptor material to be 10% of the total volume magnetic material and 2.5% for the eddy current case. The power losses per unit volume of reactor are inversely proportional to the reactor radius, whereas the heating power is constant per unit volume, resulting in increasing efficiency at higher scale and predicted efficiency of greater than 90% for reactors of 0.5 m radius or larger. However, the resonant tank circuit does constrain the frequency, and for a given power generation system, this gives a maximum effective scale for the induction heated reactor. Taking 11 kV as the maximum voltage, Figure 11 show that the required volume fraction of heating material rapidly increases below 5 kHz, limiting the reactor size to less than 0.2 m and the maximum possible efficiency for either magnetic hysteresis or eddy current heating to 65%. While further optimization of the coil design or the susceptor material may lead to further efficiency improvements, the scale of an induction heated reactor driven by a resonant tank circuit is fundamentally limited by the maximum practical voltage limit for the circuit components.

### 3.4 Discussion on Further Efficiency Improvements

Present RF generators cannot exceed 11 kV and this means that only lower frequencies can be used at larger scale. If voltage were not a limiting factor then frequency could be increased independently of reactor scale. In that case, results show that efficiency stabilizes at a maximum value above 10 kHz for a given reactor radius (Figure 13), exceeding 80% for reactor radiuses larger than 0.2 m. Alternative means of generating the applied field may result in greater energy efficiency. The Alexanderson alternator and Bethenod-Latour alternator are both capable of producing frequencies in excess of 100 kHz, at a power of 500 kW with efficiencies up to 82%.^39^ Applying 21st century advancements in materials, power generation technology and rotating equipment design to a low frequency rotating alternator could result in electricity to applied field efficiencies in excess of 90% following the bounding efficiency line in Figure 13.

Further energy efficiency gains could be made by recycling the waste heat generated in the work coil. A side-benefit of locating the work-coil inside the reactor is that the waste heat is available at higher temperature compared to the external coil. For example, an ethanol dehydration reactor operating at 50% energy efficiency could recover all of its waste heat in order to vaporise the feed stream.

## 4 Conclusions

In this paper, models have been developed for the energy efficiency of an RF induction heated reactor, incorporating the heating power of a bed of electrically conductive or magnetic material. These models include loss terms for the work coil and vessel shell and correct for the effects of nonideal applied field strength associated with the finite length of the coil. The effects of scale have been assessed by reducing the various geometrical parameters to functions of the reactor radius and aspect ratio, resulting in two sets of equations that explicitly articulate the effects of the various design and operating parameters on the efficiency of such systems. These equations imply that the efficiency of induction heated reactors continuously increases with increasing scale.

This model was applied to study both eddy current and hysteresis heating for an ethanol to ethylene model reaction, and demonstrated that the energy efficiency of these reactors can approach greater than 90% as the radius of the reactor increases toward the metre scale. Location of the work-coil is shown to be highly efficient and presents an effective route to a practical reactor design. By introducing constraints on the maximum heat generated in the coil, the design is further constrained, leading to a result that the volume fraction of heating material needed and the energy efficiency of this class of reactors is approximately uniform above 25 kHz. The relative magnitude of each energy loss term is also independent of the heating material and the reactor size. The main contribution to energy loss in the examples is the DC resistance and proximity effect in the coil, suggesting that the efficiency can be approximated without incorporating the vessel wall losses and skin effect.

The present maximum voltage limit of a resonant tank circuit limits the maximum theoretical energy efficiency. For a coil with a maximum voltage of 11 kV, the maximum energy efficiency in the ethanol-to-ethylene reaction example is 65% with a reactor diameter of 0.2 m. Alternative methods of producing a high power, low frequency field could increase efficiency in excess of 90%, such as a reintroduction of 10 kHz power alternators.

Although experimental studies will be necessary to confirm these model results, these findings taken together would indicate that RF induction heating is a highly energy efficient and practical technology for applications in industrial chemical catalytic reactor design.

## Data Availability

Data presented within this paper are available through the Bath University repository (https://doi.org/10.15125/BATH-01417).

## Supporting Information Available

Further details of the development of model including treatment of work coil losses including the skin and proximity effects and determination of electromagnetic field strength (PDF)

## Nomenclature

*A*: Arctangent magnetic model parameter, A·m^–1^
*A**: Tube annulus parameter, –
*a*: Reactor aspect ratio (length divided by diameter), –
*C*: Capacitance of resonant tank circuit, F
*F*_*layer*_: Coil layer factor, –
*F*_*hys*_: Hysteresis heating power factor, –
*F*_*prox*_: Work coil resistance proximity effect factor, –
*F*_*skin*_: Work coil resistance skin effect factor, –
*F*_*sph*_: Sphere heating power factor, –
*F*_*tube*_: Skin effect parameter associated with hollow tubes, –
*F*_*vessel*_: Vessel power factor, –
*f*: Operating frequency, Hz
*f*_*res*_: Resonant frequency of the resonant tank circuit, Hz
*H*: Applied field strength, A·m^–1^
*H*_*c*_: Coercivity, A·m^–1^
*Ĥ*: Peak applied field strength within the work coil, A·m^–1^
*Ĥ*_*ext*_: Peak applied field strength immediately outside the work coil, A·m^–1^
*Ĥ*_∞_: Applied field strength along the centerline of an infinitely long solenoid, A·m^–1^
*Î*: Peak current in the work coil, A
*K*: Applied field correction factor, –
*k*: ratio of wire spacing to wire diameter, –
*k*_*ins*_: Thermal conductivity of insulation layer, W·m^–1^·K^–1^
*L*_*a*_: Inductance of a real coil, H
*L*_*eq*_: Equivalent inductance of the combined work coil and heated bed, H
*L*_∞_: Inductance of an infinitely long solenoid, H
*l*: Length of reactor, m
*l*_*w*_: Length of the work coil circuit, m
*M*: Magnetisation, A·m^–1^
*M*_*s*_: Saturation magnetization, A·m^–1^
*m*: Number of coil layers, –
*m*_*s*_: Equivalent magnetic moment of a sphere in an applied magnetic field, A·m^–1^
*N*: Number of coil turns, –
*n*: Number of coil turns per meter, m^–1^
*P*: Power, W
*P*_*coil*_: Power dissipated as heat in the work coil, W
*P*_*ins*_: Heat loss across an external insulation layer, W
*P*_*vessel*_: Power loss due to eddy currents induced in the external metal shell of the reactor, W
*p*: Efficiency parameter, –
*p*_*eddy*_: Efficiency parameter for a bed of electrically conductive spheres, –
*p*_*flux*_: Limiting heat flux across work coil surface, W·m^–2^
*p*_*hys*_: Efficiency parameter for a bed of magnetic material, –
*R*_*DC*_: Direct current resistance of the work coil circuit, Ω
*r*: Reactor radius, m
*r*_*i*_: Tube inner radius, m
*r*_*s*_: Heating sphere radius, m
*r*_*w*_: Radius of the work coil wire or outer radius of work coil tube, m
*r*_*x*_: Effective radius of surface for heat transfer out of the work coil, m
*V*: Reactor bed volume, m^3^
*V̂*: Maximum voltage allowable in the work coil circuit, V
*w*: Arctangent magnetic model parameter, A·m^–1^
*x*_*c*_: Arctangent magnetic model parameter, A·m^–1^
*y*_*0*_: Arctangent magnetic model parameter, –
*γ*_*s*_: Sphere skin depth parameter, –
*γ*_*w*_: Wire skin depth parameter, –
*γ*_*v*_: Vessel skin depth parameter, –
*δ*_*s*_: Sphere skin depth, m
*δ*_*v*_: Vessel skin depth, m
*δ*_*w*_: Wire skin depth, m
ε: Volume fraction of heating material in the reactor, –
η: Efficiency, useful heating power per unit power supplied to the induction heater, –
Φ̂: Peak magnetic flux, Wb
λ: Wavelength of operating frequency, m
*μ*_*0*_: Permeability of free space, 4π × 10–7 H·m^–1^
μ_*r*_: Relative permeability of material, –
ν: Rayleigh parameter, m·A^–1^
*σ*_*s*_: Electrical conductivity of heating spheres, S·m^–1^
*σ*_*v*_: Electrical conductivity of vessel, S·m^–1^
*σ*_*w*_: Electrical conductivity of work coil material, S·m^–1^
χ: Susceptibility of heated bed, –
*χ*_*eddy*_: Susceptibility of bed of electrically conductive spheres, –
*χ*_*hys*_: Susceptibility of bed of magnetic material, –
*χ*_*r*_: Rayleigh’s law tip susceptibility, –
