# Supplementary material for: Radiofrequency Induction Heating for Green Chemicals Manufacture: A Systematic Model of Energy Losses and a Scale-Up Case-Study
Source: ACS Eng Au. 2024 Jul 25;4(5):450–63. doi: 10.1021/acsengineeringau.4c00009 (PMC11487564; doi:10.1021/acsengineeringau.4c00009)
Supplement: Supplementary file 1 — eg4c00009_si_001.pdf [file eg4c00009_si_001.pdf]

Radio-Frequency Induction Heating for Green Chemicals Manufacture: A Systematic Model of Energy Losses and a Scale-up Case-Study

Jonathan P. P. Noble, Simon J. Bending, and Alfred K. Hill **

Centre for Sustainable and Circular Technologies
University of Bath
The Avenue, Claverton Down, Bath, BA2 7AY, United Kingdom
E-mail: a.k.hill@bath.ac.uk; jon.noble.ceng@gmail.com

Supplementary Information

Derivation of the AC work coil power losses per unit volume:

The AC losses in the work coil are related to the DC resistance by the skin and proximity factors:

$$P_{coil} = \frac{1}{2} R_{DC} \left(F_{skin} + F_{prox}\right) \cdot \hat{I}^2 \qquad \textit{Eqn. 1}$$

$$R_{DC} = \frac{l_w}{\pi r_w^2 \sigma_w} \qquad \textit{Eqn. 2}$$

The work coil length, $l_{w,}$ is evaluated as a number of helical coils with an axial wire to close the circuit (Eqn 3 and Figure S1).

$$l_w = \left(l + \sqrt{l^2 + (2\pi r N)^2}\right) m \approx 2\pi r N m \approx 4\pi r^2 n m a \qquad \textit{Eqn. 3}$$

In most cases the term *2πrN* is significantly larger than the work coil length and so the work coil length can be approximated as a number of stacked loops of wire.

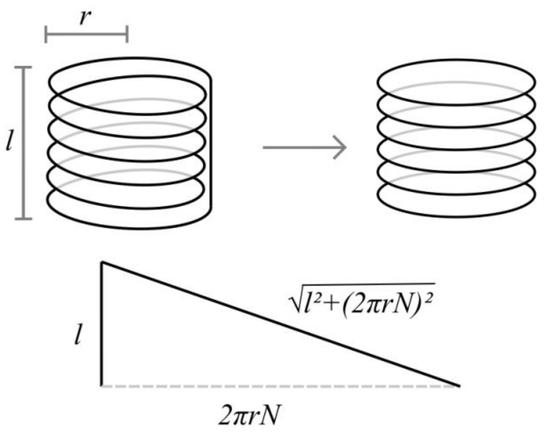

Figure S1. Visualisation of work coil conductor length. The total circuit length is the hypotenuse formed by unwrapping the coil helix plus the return leg used to close the circuit. Under most practical circumstances, the work coil can be approximated as a number of stacked circular loops (Eqn 3)

The AC losses per unit volume can therefore be expressed as:

$$\frac{P_{coil}}{V} = \frac{1}{2} R_{DC} \left(F_{skin} + F_{prox}\right) \cdot \hat{I}^2 \cdot \frac{1}{2\pi r^3 a} = \frac{nm}{\pi r\, r_w^2 \sigma_w} \left(F_{skin} + F_{prox}\right) \cdot \hat{I}^2 \qquad \text{Eqn. 4}$$

Skin Effect Parameter, F_{skin}, in solid wires

The depth that a flowing alternating current penetrates into a wire is a function of the skin depth, δ_w (Eqn 5), itself a function of frequency and wire electrical conductivity, σ_w. The skin depth parameter, γ_w, is the ratio of wire radius to skin depth scaled by a factor of $\sqrt{2}$. The work coil material is assumed to be non-magnetic and so has a relative permeability equal to one. The increase in effective resistance of a wire due to the skin effect, F_{skin}, has an analytical solution from Maxwell's electromagnetic equations. These are expressed from the real part of zero order Kelvin functions, which in turn are the real (*ber*) and imaginary (*bei*) components of the zero order Bessel function of the first kind, J_0, with an argument of $xe^{i.3\pi/4}$.[1] This can be simplified by using the first terms of the Taylor series at low frequency (the small argument limit), and through the approach to an asymptote in the high limit. At low frequencies, when the skin depth is larger than the wire diameter, current flows across the full wire cross-section and the skin parameter is equal to one, which is equivalent to the DC resistance. The skin effect parameter, F_{skin}, is an increasing function of γ_w, and is directly proportional to it at very high frequencies.

$$\delta_w = 1/\sqrt{\pi \mu_0 f \sigma_w} \qquad \text{Eqn. 5}$$

$$\gamma_w = \sqrt{2}\frac{r_w}{\delta_w} = r_w \sqrt{2\pi \mu_0 f \sigma_w} \qquad \text{Eqn. 6}$$

$$F_{skin} = \frac{\gamma_w}{2} \cdot \frac{\mathrm{ber}(\gamma_w)\mathrm{bei}'(\gamma_w) - \mathrm{bei}(\gamma_w)\mathrm{ber}'(\gamma_w)}{\mathrm{ber}'^2(\gamma_w) + \mathrm{bei}'^2(\gamma_w)} \qquad \text{Eqn. 7}$$

$$\lim_{\gamma_w \leq 2} F_{skin} \approx 1 \qquad \text{Eqn. 8}$$

$$\lim_{\gamma_w \to \infty} F_{skin} = \frac{\gamma_w}{2\sqrt{2}} \qquad \text{Eqn. 9}$$

Skin Effect Parameter in Hollow Tubes

In a hollow tube with outer radius r_w and inner radius r_i, there is a complex interaction between the tube geometry and the skin effect. The exact form of the skin parameter for a hollow tube is:[1]

$$F_{skin,\,tube} = \frac{\gamma_w}{2}.Re\left[\frac{\mathrm{ber}(\gamma_w) + i.\mathrm{bei}(\gamma_w) - \mathrm{F}_{tube} \times (\mathrm{ker}(\gamma_w) + i.\mathrm{kei}(\gamma_w))}{\mathrm{bei}'(\gamma_w) - i.\mathrm{ber}'(\gamma_w) - \mathrm{F}_{tube} \times \left(\mathrm{kei}'(\gamma_w) - i.\mathrm{ker}'(\gamma_w)\right)}\right] \times \left[1 - \left(\frac{r_i}{r_w}\right)^2\right] \qquad \textit{Eqn. 10}$$

$$\mathrm{F}_{tube} = \left[\frac{\mathrm{bei}'\left(\frac{r_i}{r_w}\gamma_w\right) - i.\mathrm{ber}'\left(\frac{r_i}{r_w}\gamma_w\right)}{\mathrm{kei}'\left(\frac{r_i}{r_w}\gamma_w\right) - i.\mathrm{ker}'\left(\frac{r_i}{r_w}\gamma_w\right)}\right] \qquad \textit{Eqn. 11}$$

$$\lim_{\gamma_w \le \sqrt{2}A^*} F_{skin,\,tube} = \frac{1}{1 - (r_i/r_w)^2} = A^* \qquad \textit{Eqn. 12}$$

$$\lim_{\gamma_w \to \infty} F_{skin,\,tube} = F_{skin} \qquad \textit{Eqn. 13}$$

The resistance of a hollow tube at low frequencies approximates to the DC resistance. At very high frequencies, the skin depth is small compared to the tube wall thickness and the AC resistance of the hollow tube converges on that of a solid wire with the same outer radius.

Combined Skin Effect Parameter

The equations in the preceding section show that the solid wire can be considered as a special case of the hollow tube in which the inner radius is equal to zero and hence A^* is equal to one. This leads to a combined result for both hollow tubes and solid wires:

$$\lim_{\gamma_w \le \sqrt{2}A^*} F_{skin} = A^* \qquad \textit{Eqn. 14}$$

$$\lim_{\gamma_w \to \infty} F_{skin,\,tube} = \frac{\gamma_w}{2\sqrt{2}} \qquad \textit{Eqn. 15}$$

Proximity Effect Factor, F_{prox}

The proximity effect factor is a function of a spacing parameter, *k,* the ratio of the wire spacing to the wire diameter.[2,3]. For a multi-turn coil made from solid wire, the proximity factor is given by Equation 16. A coil layer factor, F_{layer}, can be defined to encompass the geometry factors associated with the coil turns and number of layers, based on the last term in Equation 16. The coil layer factor is equal to *12π* for a single layer coil and rapidly approaches 16π for coils with two or more layers. The limits of the Bessel functions are given at low and high skin depth ratios in Equations 19 and 20.

$$F_{prox} = -\,\gamma_w.\frac{\mathrm{ber}_2(\gamma_w)\mathrm{ber}'(\gamma_w) + \mathrm{bei}_2(\gamma_w)\mathrm{bei}'(\gamma_w)}{\mathrm{ber}^2(\gamma_w) + \mathrm{bei}^2(\gamma_w)} \cdot \frac{\pi^2(4m^2 - 1)}{12k^2} \qquad \textit{Eqn. 16}$$

$$k = \frac{1}{2r_w n} \ge 1 \qquad \textit{Eqn. 17}$$

$$F_{layer} = 4\pi\left(4 - \frac{1}{m^2}\right) \qquad \textit{Eqn. 18}$$

$$\lim_{\gamma_w \leq \sqrt{2}} F_{prox} \approx \frac{\gamma_w^4}{192} \pi r_w^2 n^2 m^2 \cdot F_{layer}$$ *Eqn. 19*

$$\lim_{\gamma_w \to \infty} F_{prox} = \frac{\gamma_w}{12\sqrt{2}} \pi r_w^2 n^2 m^2 \cdot F_{layer}$$ *Eqn. 20*

Relating the Coil Current to the Applied Field Strength

The applied field strength is dependent on the work coil current and geometry. It can be derived from the Biot-Savart law using an analytical solution for the magnetic field associated with the current flowing in a circular loop, and a long coil is approximated to be a number of stacked loops. The internal magnetic field is the sum of the magnetic fields and gives a uniform magnetic field strength within an ideal solenoid (H_∞).

$$\hat{H}_\infty = nm\hat{I}$$ *Eqn. 21*

Equation 21 is valid for an ideal coil, but for a real coil end effects reduce both the axial and radial field strength. The reduction along the central axis is given in Equation 28 and results are presented in Figure S2 for coils of different aspect ratios, *a*, where *x* is the axial distance from the dead-centre of the coil.[4] The axial field strength within the coil appears reasonably uniform for both low and high coil aspect ratios.

$$\frac{\hat{H}_x}{\hat{H}_\infty} = \frac{1}{2}\left[\frac{a + x/r}{\sqrt{1 + \left(a + x/r\right)^2}} + \frac{a - x/r}{\sqrt{1 + \left(a - x/r\right)^2}}\right]$$ *Eqn. 22*

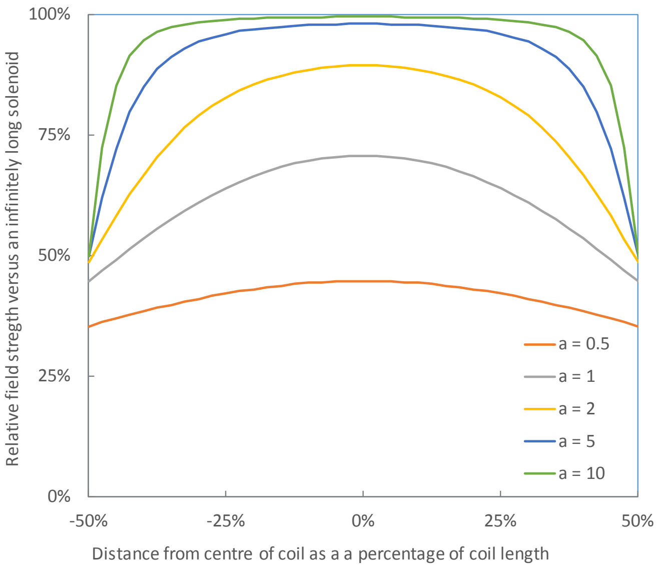

Figure S2: The axial field strength down the centreline of a cylindrical solenoid for short coils with differing length to diameter ratio (aspect ratio, a), relative to an infinitely long solenoid of the same radius. Calculated from Eqn 22.

The inductance of a coil,. The applied field correction factor, K, is the ratio of the real inductance over the inductance of an infinitely long coil, L_∞ (Eqn 24). It is calculated from the coil inductance, L_a, given in Equation 23 and it allows for the field strength produced by a real coil to be predicted. The mutual inductance between layers in a multilayer coil are neglected because the coil thickness is small compared to its radius. The average

coil layer radius is approximately constant and the effects on the inductance can be considered solely as an increase in the number of turns per metre of coil.

$$L_a = Nm\frac{\hat{\Phi}}{\hat{I}} = Nm\mu\pi r^2\frac{\hat{H}}{\hat{I}} = 2\pi\mu_r\mu_0 r^3 anm\frac{\hat{H}}{\hat{I}} \qquad \text{Eqn. 23}$$

$$L_\infty = \frac{\mu N^2 m^2 \pi r^2}{l} = \mu_r\mu_0 n^2 m^2 \cdot 2\pi r^3 a \qquad \text{Eqn. 24}$$

$$\hat{H} = \frac{L_a}{2\pi\mu_0 r^3 an}\hat{I} = \frac{L_a}{L_\infty}nm\hat{I} = Knm\hat{I} \qquad \text{Eqn. 25}$$

Wheeler's formula[5] provides a simple relationship for the applied field correction factor, *K*, with a maximum relative error of 1.7%.

$$K = \frac{L_a}{L_\infty} = \frac{2a}{\pi}\left(0.48.ln\left(1+\frac{\pi}{2a}\right) + 0.52.asinh\left(\frac{\pi}{2a}\right)\right) \qquad \text{Eqn. 26}$$

The applied field correction factor, *K,* is an increasing function of the aspect ratio, exceeding 0.7 at an aspect ratio of one and asymptotically approaching unity for higher aspect ratios.

References

1. Meredith, R. J. *Conduction and Induction Heating*; The Institution of Electrical Engineers, London 1991; Vol. 5., 2 https://doi.org/10.1049/pe:19910014.
2. Ferreira, J. A. Improved Analytical Modeling of Conductive Losses in Magnetic Components. *IEEE Trans. Power Electron.* **1994**, *9* (1), 127–131. https://doi.org/10.1109/63.285503.
3. Bartoli, M.; Noferi, N.; Reatti, A.; Kazimierczuk, M. K. Modeling Litz-Wire Winding Losses in High-Frequency Power Inductors. *PESC Rec. - IEEE Annu. Power Electron. Spec. Conf.* **1996**, *2* (2), 1690–1696. https://doi.org/10.1109/PESC.1996.548808.
4. Jiles, D. *Introduction to Magnetism and Magnetic Materials,* Chapman and Hall; London 2015. https://doi.org/10.1201/b18948
5. H. A. Wheeler. Inductance Formulas for Circular and Square Coils. *Proc. IEEE* **1982**, *70* (12), 1449–1450.
